# Context-Dependent Links between Song Production and Opioid-Mediated Analgesia in Male European Starlings (*Sturnus vulgaris*)

**DOI:** 10.1371/journal.pone.0046721

**Published:** 2012-10-02

**Authors:** Cynthia A. Kelm-Nelson, Sharon A. Stevenson, Lauren V. Riters

**Affiliations:** Department of Zoology, University of Wisconsin-Madison, Madison, Wisconsin, United States of America; Rutgers University, United States of America

## Abstract

Little is known about the neural mechanisms that ensure appropriate vocal behaviors within specific social contexts. Male songbirds produce spontaneous (undirected) songs as well as female-directed courtship songs. Opioid neuropeptide activity in specific brain regions is rewarding, at least in mammals, and past studies suggest that the opioid met-enkephalin in such areas is more tightly linked to undirected than female-directed song. Recent data using a song-associated place preference paradigm further suggest that production of undirected but not directed song is tightly linked to intrinsic reward. Opioids have analgesic properties. Therefore, if production of undirected song is closely linked to opioid-mediated reward, the production of undirected but not directed song should be associated with analgesia. Consistent with this prediction, in male starlings we identified a positive correlation between analgesia (decreased reactivity to a hot water bath) and undirected song (in non-breeding season condition males in affiliative flocks) but not female-directed song (in breeding season condition males presented with females). When breeding condition males were divided according to social status, a negative correlation was found in subordinate males (i.e. males that failed to acquire a nest box). These data are consistent with the hypotheses 1) that the production of undirected song is facilitated or maintained by opioids (and/or other neuromodulators that also induce analgesia) and 2) that production of female-directed song is not linked in the same way to release of the same neuromodulators. Results also demonstrate a link between analgesia and song in subordinate individuals lacking a nesting territory within the breeding season. Overall, the findings indicate that distinct neural mechanisms regulate communication in different social contexts and support the working hypothesis that undirected but not directed song is tightly linked to opioid release.

## Introduction

Vocal communication plays a critical role in social interactions across vertebrate species, including songbirds [1]. To communicate effectively individuals must adjust vocal production to match particular social contexts, yet little is known about neural mechanisms underlying context-appropriate communication.

In songbirds, opioid neuropeptides are proposed to play a role in male singing behavior that differs depending upon whether song is produced spontaneously (undirected) or is sexually-motivated and directed towards a female (female-directed song). Specifically, immunolabeling density for the opioid met-enkephalin in the medial preoptic nucleus (referred to as POM in birds) correlates positively with undirected but not female-directed singing behavior in male European starlings (*Sturnus vulgaris*), with a similar trend observed in the ventral tegmental area (VTA; p = 0.06) [2]. Additionally, mu-opioid receptor labeling density is lower in both of these regions in male starlings singing high compared to those singing low rates of female-directed song [3]. Pharmacological manipulations in male starlings and zebra finches (*Taeniopygia guttata*) also indicate that opioids regulate song differently depending on whether it is female-directed or undirected [2], [4], [5]. In rats, opioid neuropeptides in VTA and the preoptic area are rewarding (e.g., morphine in VTA and enkephalin in the preoptic area [6], [7]), and recent data in starlings using a song-associated place preference paradigm suggest production of undirected but not directed song is tightly linked to reward state [8]. Together these studies lead to the hypothesis that undirected song is more tightly linked to immediate opioid release in the POM and VTA than directed song (reviewed in [9], [10]).

Opioids have analgesic properties [11], [12], and data indicate that opioid release in both the preoptic area and VTA induces analgesia in rats [13], [14]. If production of undirected song is regulated by immediate opioid release in these regions, then production of undirected but not directed song may be associated with analgesia. To test this prediction, flocks of male starlings were observed singing undirected song (males with low testosterone (T) singing in an affiliative non-sexual context) and female-directed song (males with high T singing to females in a breeding season condition). Immediately after the observation period, the latency for each male to remove his foot from a hot water bath was recorded as a measure of analgesia. If undirected but not directed singing behavior is regulated by immediate opioid release, we predicted that measures of undirected but not directed song would correlate positively with the length of time a male maintained a foot in hot water.

## Materials and Methods

### Ethics Statement

Protocols used for bird acquisition, housing, and behavioral testing were in adherence to guidelines approved by the *National Institutes of Health Guide for the Care and Use of Laboratory Animals* (DHEW Publication 80–23, Revised 1985, Office of Science and Health Reports, DRR/NIH, Bethesda, MD 20205). The studies described here were approved by the University of Wisconsin-Madison Institutional Animal Care and Use Committee (Protocol Number: L00379-0-08-06).

### Animals

In November and December of 2008 and 2009, 81 adult male European starlings (*Sturnus vulgaris*) and 10 adult females were captured on a single farm west of Madison, WI using baited fly-in traps (Table 1). A Federal Migratory Bird Scientific Collecting permit is not required for European starlings as they are not covered under the Migratory Bird Treaty Act. After capture, birds were housed indoors in the University of Wisconsin-Madison Department of Zoology animal facilities in single sex cages (91 cm × 47 cm ×47 cm) on photoperiods similar to the outdoor natural light cycle. Food (Purina Mills Start and Grow Sunfresh Recipe, 61S3-IGH-G) and tap water were always provided ad libitum. Each animal was assigned a number as well as a colored leg band for identification.

**Table 1:** Final sample sizes for each experiment.

Experiment	Number of Animals
1. Latency to withdrawal curve	9
2. Test to confirm thermal test is opioid sensitive	15
3a. Song Associated Analgesia: Breeding Condition 3b. Analysis of behaviors, T and analgesia*	35 20
4. Song Associated Analgesia: Non-Breeding Condition	22

* subset of the same birds used in 3a.

### Analgesia Test

The analgesia test described here is similar to an opioid-sensitive analgesia test used in past work on Japanese quail (*Coturnix japonica*) [15] and house sparrows (*Passer domesticus*) [16]. A 250 ml beaker was filled with tap water and placed on a hot plate. The water was stirred and the temperature was measured continuously using a digital thermometer with a resolution of 0.1°C (Traceable Thermometer, −50°C to 150°C). Each subject was held in one hand and the foot up to the ankle joint was quickly lowered into the water bath. The head was covered with a hood to reduce visual distraction. *Foot withdrawal latency* (i.e., analgesia) was measured as the time for the bird to remove its foot from the water bath in a temperature established in Experiment 1. Times were recorded using a stopwatch with a resolution of 0.01 s (ThermoScientific: Cimarec). The maximum time allowed for testing was 20 s; thereafter the foot was manually removed. The ambient air temperature of the room was within the range of 20–26°C. After the test, the foot was submerged in room temperature water and the animal returned to its home cage.

### Experiment 1: Latency to Withdrawal Curve

Nine (Table 1) birds were housed in groups of three on 8 hours of light (L): 16 hours of darkness (D) in single sex cages and were used to identify the temperature that generated a measurable analgesia response. The foot withdrawal latency for each bird was tested at water temperatures ranging from 40°C to 60°C in increments of 2.5°C [16]. The order of water temperatures tested was randomly selected for each bird, to avoid confounding group and day effects. At least one full day was used as a recovery period between testing (testing did not exceed three times in a single week). Testing was performed in the light phase between 8∶30 and 11∶30. The total procedure time, from capture to returning a bird to its standard home cage, lasted less than five minutes per trial. Each male was tested a total of nine times.

### Experiment 2: Test to confirm the thermal test is opioid sensitive

In this experiment we examined whether peripheral injections of deionized water (vehicle control), naloxone (opioid antagonist, 20 mg/kg dissolved in deionized water), or fentanyl (opioid agonist, 0.25 mg/kg dissolved in deionized water) modify the foot withdrawal response to hot water.

Males, n = 15 (Table 1), were housed on artificial photoperiods of 18L: 6D for a length of six weeks, a photoperiod which induces refractoriness, a physiological state observed naturally in starlings in late summer/early fall in which the level of circulating reproductive hormones is basal [17]. This photoperiod was selected to mitigate any possible effects of steroid hormones on analgesia. Animals were housed in groups of 5 in single sex cages (91 cm ×47 cm ×47 cm). Each male was injected with 0.05 mL (subcutaneous injection, inguinal leg fold) of the appropriate dose of the drug (5 birds per drug group), and placed in a covered holding cage (birds were visually but not acoustically isolated). Thirty minutes after injection, analgesia was measured in each male using the hot water test described above. Time after injection was designed to fit within the half-life of the drug provided by the manufacturer. The latency to withdrawal was averaged in each group.

### Experiment 3: Tests of Song-associated Analgesia

#### Breeding Season Condition

Photoperiod and hormone manipulations were used to place males into a physiological state characteristic of the natural spring breeding season. Specifically, birds were placed on photoperiods of 18L: 6D for 6 weeks, followed by 6L: 18D for an additional 6 weeks. Exposure of male starlings to this regime of long followed by short photoperiods induces a physiological state referred to as photosensitivity, a condition in which males respond to increasing day length (characteristic of the spring breeding season) and testosterone with increases in the production of sexually-motivated behaviors, including courtship song [3], [17], [18]. Birds were moved into single-sex indoor aviaries (3.5 m ×2.25 m ×2 m) on photoperiods of 11L: 13D, a photoperiod under which male starlings respond to testosterone treatment with increases in female-directed singing behavior (e.g., [3], [19]). Birds were randomly assigned to groups ranging from two to five. Aviaries contained nest boxes, multiple perches, food and water. Aviaries were visually isolated.

Each breeding season condition male, n = 35 (Table 1), received two silastic subcutaneous implants of T (two, 14 mm in length of i.d., 1.47 mm; o.d. 1.96 mm; Dow Corning, Midland, MI USA, packed with 10-mm crystalline testosterone proprionate, Sigma-Aldrich, St. Louis, MO USA). Each stimulus female, n = 10, received two silastic implants of 17β-estradiol (two, 17 mm in length packed with 13 mm 17β-estradiol, Sigma-Aldrich), to enhance female sexual interest. Hormone implants were surgically placed above the left breast muscle two weeks prior to behavioral testing as described in [3]. Past results show that the T manipulation in males elevates serum T concentrations to those observed within the breeding season [20].

Immediately prior to a single behavioral observation period, a novel stimulus female and nest material (green grass clippings and leaves) were introduced into the aviary. Male singing behavior was observed for 20 minutes between 10∶00 and 14∶30 hours. Starling song consists of four components: introductory whistles, complex phrases, click series and high frequency phrases [21]. An observer located behind a one-way mirror recorded the number of times a starling produced any component of song. A song was considered new if separated by at least 2 seconds. Components were summed to create a measure of *Total Song*. In addition, gathering nest material and the number of nest box entries was also recorded.

Immediately after the 20 min observation period a second experimenter entered the room and rapidly captured the subject. The right foot of the subject was placed in a water bath containing water at 55°+/−0.3°, a temperature established in experiment one, and the *Latency to Withdrawal* the foot was recorded.

In order to determine whether the analgesia measure can be explained by behaviors other than song, for a subset of animals (n = 20; Table 1), additional measures of behavior were collected, specifically feeding and drinking. A distinct bout of behavior was defined as an event separated from the next event by at least 2 seconds. Additionally, after the testing period, each male was checked to confirm the presence of hormone implants and a blood sample was taken for T analysis. A single sample of 200 μL of blood was collected via venipuncture of the ulnar vein of 19 males. Plasma T was measured using a competitive immunoassay (EIA; Cayman Chemical, Ann Arbor, MI, USA, Catalog No. 582701) in accordance to manufacturer's directions and as reported in [3].

#### Non-breeding Season Condition

Males, n = 22 (Table 1), in the non-breeding season condition were placed on photoperiods of 18L: 6D for six weeks followed by a photoperiod of 6L: 18D for the remainder of the study. As described above, this photoperiod regime induces photosensitivity. Under natural conditions male starlings become photosensitive in the fall non-breeding season, and as long as day length is relatively short (throughout the fall and winter non-breeding season), testosterone remains low [17], [22]. Males were housed in groups of four in aviaries set up as described for the breeding season condition males. Birds were observed behaviorally and tested for withdrawal response as described above. However, a female and nest material were not introduced into the aviary as these are not biologically relevant stimuli for male starlings in a non-breeding condition [23]. Measures of eating and drinking were also recorded. T was not measured in males in this condition because based on past studies the circulating levels of T are below the detectable level of the assay (e.g. [24]).

### Statistical Analysis

All data were analyzed using Statistica 6.0 software (Stat Soft Inc., Tulsa, OK). Levene's test for homogeneity of variance and Lilliefors test for normality were used to test the assumptions required for the use of parametric statistics. Outliers identified in residual analysis plots were removed if they fell outside two times the standard deviation of the mean. This resulted in the removal of a single breeding season condition male from the song-associated analgesia study (latency to withdraw foot  = 19.42 sec, total song = 1) and one male from the pharmacology study (latency to retract foot  = 20 sec after each manipulation). (These outliers are not included in sample sizes described above or in Table 1).

### Peripheral Pharmacology

A one-way analysis of variance (ANOVA) was used to examine differences in the foot withdrawal response across pharmacological treatments. Post-hoc analyses of significant ANOVA results were performed using Fisher's LSD tests.

### Song-associated Analgesia

Pearson correlations were used to evaluate relationships between latency to withdrawal the foot and total song in the breeding and non-breeding season conditions. The relationship between the analgesia response and additional measures (i.e., feeding and drinking, plasma T) were also analyzed using Pearson correlations for the subset of breeding season condition birds for which these measures were collected.

Within the breeding season condition some males acquired nesting territories (nest boxes); others did not. In starlings nest box owners sing high rates of song in response to the introduction of a female (female-directed song) and socially dominate other males [23], [25]. Males without boxes sing, but do not increase their rates in the presence of a female and are socially subordinate to other males [23], [25]. Based on these behavioral differences we also analyzed data in males with and without nest boxes separately. Nest box owners were defined as males entering and exiting the same nest box at least 2 times during the observation period.

In all conditions, birds that did not sing during the behavioral observation period were dropped from analysis (non-breeding season condition n = 6, breeding season condition n = 9 (1 nest box owner, 8 non-owners)). (Birds that did not sing are not included in sample sizes described above or in Table 1).

## Results

### Latency to withdrawal

**Figure 1:**
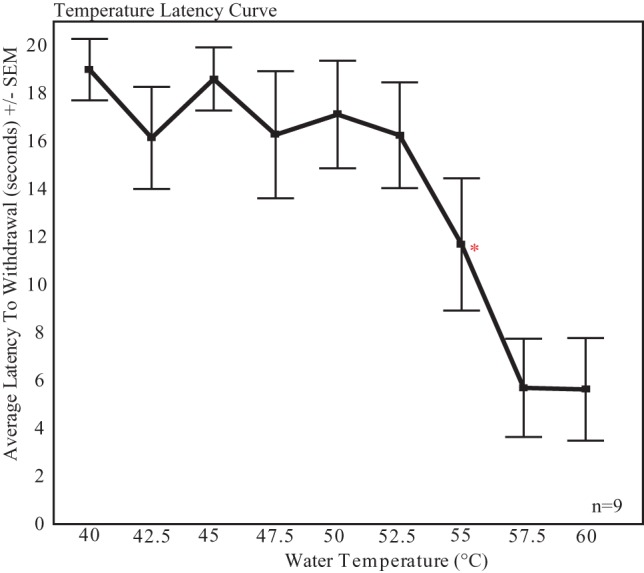
Temperature latency curve. The curve showing mean (+/− standard error) latency to withdrawal the foot at increasing water temperatures in male starlings. The temperature of 55.5°C (indicated with an asterisk) was selected for all experiments as it fell within the slope of the curve and generated a measurable response in all subjects.

The mean latency +/− the standard error at each temperature (40–60°C) was plotted to establish a temperature latency curve (Figure 1). Similar to Japanese quail [15] and house sparrows [16], the latency curve had a sigmoid shape with a “non-response plateau” at low temperatures, a “high response plateau” at high temperatures and a slope. The temperature of 55°C+/−0.3°C was selected as it fell within the slope portion of the curve and generated measureable responses.

### Peripheral Pharmacological Manipulations

**Figure 2:**
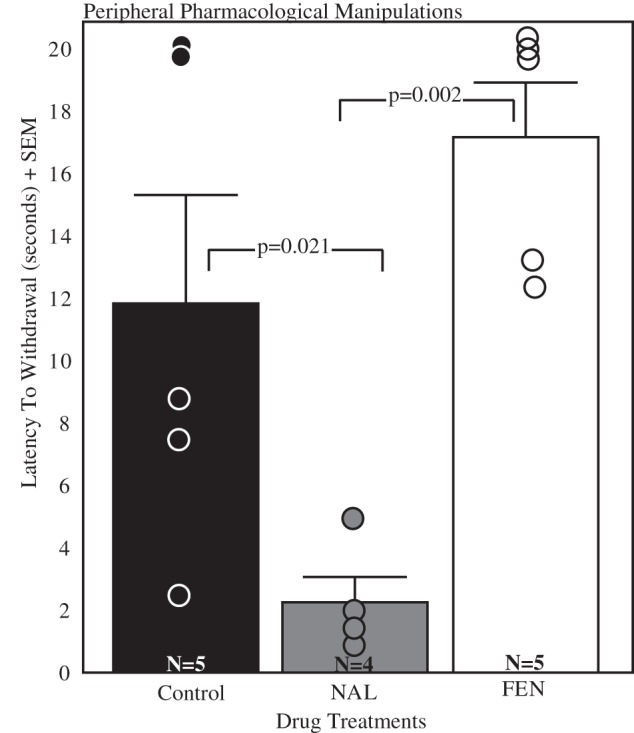
The foot withdrawal response is opioid sensitive. Mean latency to withdrawal and standard error in males receiving peripheral injections of diH_2_0 (control; black bar); 20.0 mg/kg naloxone (NAL; gray bar); 0.25 mg/kg fentanyl (FEN; white bar). Individuals are represented by a single circle in each condition. Sample sizes are indicated in the bottom of each bar. Brackets indicate the results of the Fisher post hoc contrasts.

A one-way ANOVA indicated that drug treatments significantly altered the analgesia response (F_(2, 11)_  = 8.85, n = 14; p = 0.005; Figure 2). Fisher LSD post hoc tests revealed significant differences between naloxone and control treatments (p = 0.021) and naloxone and fentanyl treatments (p = 0.002). There was no significant difference between fentanyl and control treatments (p = 0.14).

### Comparisons of Breeding and Non-Breeding Season Condition Birds

**Figure 3:**
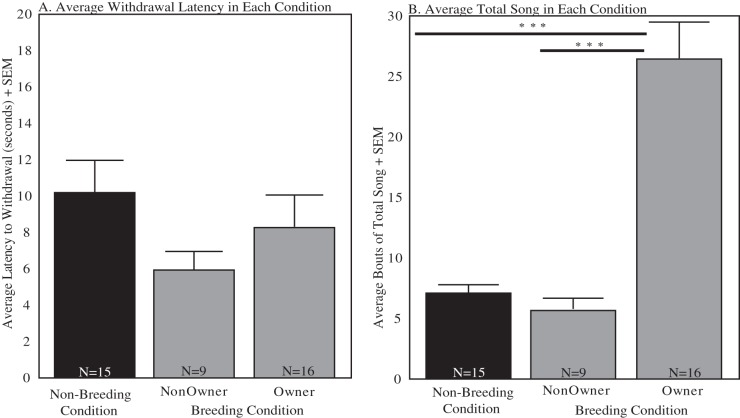
The average latency to withdrawal the foot did not differ across groups. The average bouts of Total Song in breeding nest box owners were significantly different from non-owners and non-breeding condition. A. Latency to withdrawal the foot in non-breeding condition (black bar) and breeding condition (nest box non-owners and owners, gray bars) +SEM. B. Average bouts of total song in each condition +SEM. Statistical significance is represented by lines and *** (*p*<.00001). Sample sizes are indicated in the bottom of each bar.

The average latency to withdrawal in breeding season and non-breeding season condition males was calculated. There were no significant differences between the two groups (mean of non-breeding = 9.54, SD = 6.83; mean of breeding = 7.43, SD = 6.05; t_38_ = 1.02, p>0.10; Figure 3A). There was also no difference when breeding condition males were analyzed based on nest box status (mean of non-owners = 5.93, SD = 3.071; mean of owners = 8.27, SD = 7.17; t_23_ = 0.92, p>0.10; Figure 3A). The average bouts of total song were also calculated in each condition. There was a significant difference in total singing behavior between the breeding season and non-breeding season (mean of non-breeding = 7.0, SD = 2.67; mean of breeding = 18.96, SD = 14.14; t_38_ = 3.23, p = 0.0026l; Figure 3B). There was also a difference between owners and non-owners (mean of non-owners = 5.67; SD = 3.04; mean of owners = 26.43, SD = 12.2; t_23_ = 4.97, p = 0.000050; Figure 3B). There was no difference between non-breeding season and non-owners (t_22_ = 1.124, p = 0.27; Figure 3B); however, there was a significant difference between non-breeding season and owners (t_29_ = 6.023, p = 0.000001; Figure 3B).

### Non-Breeding Season Condition

**Figure 4:**
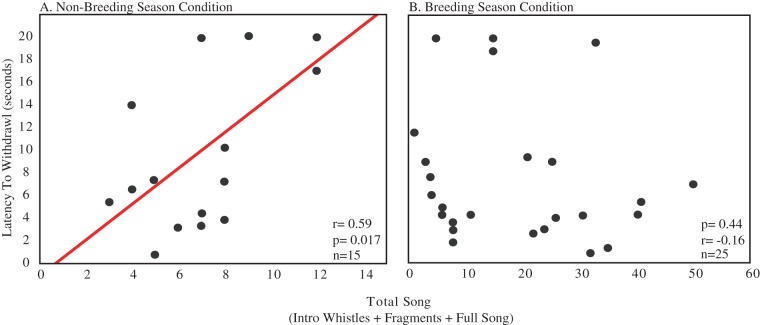
Analgesia responses correlate with song production in non-breeding season condition males. Data shown are the latency to withdrawal the foot (in seconds) versus the total song produced by each individual. Each point represents one individual. A. Individuals in the non-breeding season condition. B. Breeding season condition individuals. Sample sizes are noted in the bottom right corner. Presence of the regression line indicates a significant correlation (*p*<0.05).

A significant positive correlation was identified between total song production and analgesia (n = 15, r = 0.59, p = 0.017; Figure 4A). There was no correlation between analgesia and the measure of feeding and drinking (n = 15, r = −0.37, p = 0.16).

### Breeding Season Condition

**Figure 5:**
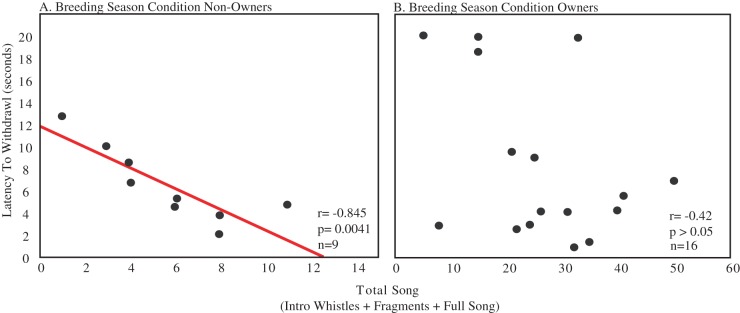
Analgesia responses within the breeding season condition correlate with song production in males without nest boxes. Data from Fig. 4B have been replotted to illustrate relationships between the latency to withdrawal the foot (in seconds) versus the total song produced by each individual for A. Breeding season condition individuals that did not occupy a nest site and B. Breeding season males that occupied and defended nest sites. Sample sizes are noted in the bottom right corner. Presence of the regression line indicates a significant correlation (*p*<0.05).

Correlation analyses revealed no significant relationship between total song and analgesia in the breeding season condition males (n = 25, r = −0.16, p>0.10; Figure 4B). However, when nest box owners (entering box mean = 3.4, SD = 5.55; gathering nest material mean = 0.9, SD = 1.80) and non-owners (no nest box directed behaviors) were analyzed separately, there was a significant negative correlation between the variables in non-owners (n = 9, r = −0.845, p = 0.0041; Figure 5A) but not owners (n = 16, r = −0.42, p = 0.11; Figure 5B).

No significant correlations were identified between analgesia and feeding and drinking (n = 20, r = 0.36, p = 0.11), in the 10 nest box owners and 10 non-owners for which these measures were collected. Furthermore, there was no correlation between T and analgesia (n = 19, r = −0.24, p>0.10). Additionally, T did not differ between nest box owners and non-owners (mean nest box owners = 3391.80 pg/mL, SD = 324.82, mean non-owners = 3339.48 pg/mL, SD = 362.87; t_17_ = 0.33, p>0.10).

## Discussion

In starlings, opioids within the POM and possibly VTA have been linked closely to undirected but not directed song production [2]. Opioid release in these regions also leads to analgesia, at least in rats [13], [14]; therefore, if production of undirected song is linked to opioid release in these areas, then production of undirected, but not directed song should be associated with analgesia. The present study supports this hypothesis and is the first to demonstrate a tight link between analgesia measures and production of undirected but not female-directed male song. Furthermore, in subordinate breeding season condition male starlings that did not defend a nest site (non-owners), analgesia and song were negatively correlated, suggesting a possible distinct role for opioids in singing behavior in this context as well.

Injections of the opioid antagonist naloxone significantly decreased analgesia compared to control injections, whereas the opioid agonist fentanyl increased analgesia compared to the naloxone treated animals. Fentanyl in the present study did not statistically increase analgesia relative to control injection, which may reflect a ceiling effect; however, our results overall are consistent with past studies showing the analgesia test used here to be opioid-dependent [15], [16].

### The production of undirected song correlated positively with analgesia

Few studies have examined opioids and undirected song, but in those that have, research suggests that opioids stimulate song in this context. For example, in zebra finches undirected song is inhibited by opioid antagonist injections [4]. Consistent with these findings, in male starlings the densities of immunolabeled met-enkephalin fibers in POM correlate positively with undirected song [2]. In addition to mediating analgesic responses, opioids (e.g., met-enkephalin and morphine) in both the preoptic area and VTA respectively is rewarding, at least in mammals [6], [7]. Undirected song is not associated with any form of obvious, immediate, external reward (e.g., it does not immediately attract a mate). Our working hypothesis is that undirected song is triggered and maintained by *intrinsic-reward* induced by release of rewarding neurochemicals such as opioids [9], [10]. Recently, the rewarding properties of producing directed and undirected song were evaluated in male starlings and zebra finches using a conditioned place preference paradigm [8]. Males of both species were found to develop a strong preference for a place associated with the act of producing undirected (but not directed) song, thereby linking the production of undirected song to a positive affective state (i.e., a reward state). Together, the analgesia results reported here along with the place preference data indicate that opioid release may underlie reward associated with undirected vocal communication. Additional research using POM and VTA site-specific pharmacological manipulations and measures of analgesia and reward are needed to evaluate this hypothesis.

### Female-directed song did not relate to analgesia

Although it is possible that some of the songs produced by males in the breeding season condition were undirected, our assumption based on past studies of males with elevated T (e.g. [23]) is that a larger proportion of the songs produced by breeding season condition males tested in the presence of a female are directed than undirected; and certainly these males produce more directed songs than non-breeding season condition birds (who based on past literature are unlikely to sing any female-directed songs) [23]. We found no correlation between the measure of analgesia and female-directed song production in dominant breeding season condition males with nesting sites, suggesting that opioid release is not linked to the production of female-directed song in the same way as undirected song. This idea is supported by past data showing that densities of immunolabeled met-enkephalin fibers in POM did not correlate linearly with directed song in male starlings [2]. Furthermore, males that occupy a nest site have a lower density of mu-opioid receptors in POM and VTA compared to males without nest sites [3]. Additionally, enkephalin opioids in the avian POM have been found to suppress male sexual behavior [26]. Thus, a reduction in opioid activity in males with nest sites should serve to facilitate sexually-motivated male behaviors, including production of courtship song. This idea is supported by data showing that peripheral administration of the opioid receptor antagonist naloxone facilitated female-directed song in male starlings [5]. In the VTA, mu-opioid receptor immunolabeling is negatively correlated with measures of total song in breeding condition starlings [3], perhaps reflecting an inhibitory role for opioids in VTA in male song in this context. In contrast to undirected song, female-directed song can result in immediate mate attraction and copulation. Thus, our working hypothesis is that directed song is primarily *externally-reinforced* by neurochemicals (including opioids) released upon successful mate attraction and copulation [9], [10] rather than in close association with the act of song production. It is also possible that opioids are released during female-directed song at low levels that are not detectable using an analgesia measure such as the latency to withdrawal from a hot water bath. Therefore, additional direct measures of opioid release such as microdialysis measures should be investigated in future studies.

### Song correlated negatively with analgesia in subordinate breeding season condition males

Here, we found a negative correlation between the measure of analgesia and singing behavior in subordinate breeding season condition males that failed to acquire nest boxes. During the breeding season, males that do not obtain nest sites sing, but they do not increase singing behavior in response to females [23]. This type of song may be a form of directed song that is suppressed in males that fail to acquire a nesting location. This idea is supported by the observation that when a nest box owner is removed from an aviary it is common for a male without a nest box to rapidly (within hours) take over the box and initiate high rates of female-directed song (personal observation). Endogenous opioids (e.g. enkephalins and endorphins) inhibit socio-sexual behaviors [27], [28]; and in starlings pharmacological manipulations indicate that mu opioid receptor stimulation inhibits female-directed song [5] (but see [4] for an exception). Male starlings without nest sites have significantly higher densities of mu-opioid receptors in the POM and VTA and other areas than males with nest sites [3]. As reviewed above, enkephalin opioids in the avian POM inhibit male sexual behavior [26]. Thus, heightened tissue sensitivity to opioids (reflected in higher receptor densities) and heightened release (reflected in the analgesia response reported here) suggest that opioids may be acting to suppress courtship song in males without a nesting site. The possibility that opioid release (and associated analgesia) serves to suppress sexual behavior in contexts in which it may not be appropriate (e.g., for a male without a nest site) is consistent with a past study in mice in which a reduction in female sexual responses to potential mates infected with parasites was associated with opioid-mediated analgesia [29].

### Analgesia did not differ categorically across conditions

Although males in spring condition with nest boxes sang at much higher rates than males in spring condition without nest boxes or fall-condition males, mean analgesia responses did not differ categorically across breeding season conditions. We do not believe that the lack of categorical differences in analgesia responses rule out our interpretations of the correlational data (that opioid release is differentially linked to communication in distinct contexts). The lack of categorical differences may reflect the fact that opioids are involved in multiple processes in addition to singing, that also differ across birds in the three conditions (e.g., feeding, stress, thermoregulation, reproductive physiology (reviewed in [11], [28], [30])). Furthermore, we expect that differences in tissue sensitivity to opioids or differences in receptor subtype distributions in males in the three conditions also may explain why a high song rate would not always be associated with high analgesia (e.g., in spring-condition birds with nest boxes) and why a low song rate would not always be linked to low analgesia (e.g., in fall-condition birds). This idea is supported by data in male dark-eyed juncos showing that mu and kappa opioid receptor densities differed seasonally in the POM and VTA [31], and data in starlings showing that mu receptor densities were greater in spring condition males with next boxes compared to those without nest boxes [3]. These findings suggest that even though birds in spring condition with a nest box sing more than birds in other conditions, opioid-mediated analgesia may not differ because the densities of opioid receptors in brain regions mediating this response differ in males across conditions. These factors may in part explain why analgesia responses do not differ categorically across groups.

### Opioids and Steroid Hormone Interactions

Steroid hormones are known to alter neural reward systems and shape behavior so that it is appropriate for an individual within a particular context. For example, in female rats proceptive behaviors alter steroid hormone levels so that copulation is rewarding [32]; pregnancy hormones are known to influence neural reward systems so that interactions with offspring are rewarding at birth [33]; and reward associated with feeding behaviors is rapidly adjusted by nutrient-induced hormone actions on reward circuitry [34]. In the present study seasonal and social status-related differences in T activity may have contributed to changes in opioid reward systems so that males sing a song appropriate for the season and an individual's social status. T strongly influences the motivation to sing in male starlings [23] and, in rodents, enkephalin opioids have been found to be affected by T, including within the preoptic area and VTA [35], [36], [37]. Data also indicate that mu opioid receptor densities shift seasonally in POM and VTA in association with testis volume [31]. It is thus possible that T differences in the present study contributed to the differential links identified between analgesia and singing behavior. Here, we found no differences in the overall analgesia responses for males in either hormonal condition. In a subset of our data, we analyzed circulating T concentrations in breeding condition males but did not see any significant relationships between T concentrations and analgesia. Previous studies indicate that T has inconsistent effects on analgesia [38], [39], including in birds using the same test employed in the present study [16], [40]. Thus, the impact of steroid hormones on opioid activity, singing, and analgesia is at present unclear.

### Future Directions and Broader Impacts

While this study focused on opioids and analgesia, there are several other neurotransmitter systems that affect analgesia and may contribute to the effects observed here, including GABA, endocannabinoids, and substance P [41], [42], [43]. Furthermore, there are several brain regions in addition to the preoptic area and VTA that regulate analgesia and contain opioid receptors, including the periaqueductal gray [44] the nucleus accumbens [45]. and the anterior hypothalamus [13], [46]. Finally, while the majority of research has focused on the mu-opioid receptor, future research should target multiple opioid receptor subtypes such as kappa and delta. The mechanisms underlying the links between singing and analgesia reported here must be identified in future work.

Human data also link undirected vocal behaviors to analgesia and opioid release. For example, swearing that was not directed toward another individual increased pain tolerance compared to tolerance in individuals that did not swear [47]. Furthermore, relaxed social laughter in humans that is considered important for group bonding (similar to undirected song in overwintering starling flocks) was associated with feelings of well-being as well as analgesia [48]. Thus, the link between analgesia and undirected vocal behavior identified here appears to extend beyond songbirds. This link may have implications for the use of vocal production in humans to promote positive affect and to reduce responses to painful stimuli in a clinical or hospital setting.
